# ETV4 Improves Cerebral Ischemia‐Reperfusion Injury by Restraining YBX1‐GPX4‐Ferroptosis Cascades

**DOI:** 10.1002/brb3.71548

**Published:** 2026-06-16

**Authors:** Qian Xu, Faming Deng, Dan Yu

**Affiliations:** ^1^ Department of Neurology Haikou Affiliated Hospital of Central South University Xiangya School of Medicine Haikou Hainan China; ^2^ Department of Emergency, Hainan General Hospital Hainan Affiliated Hospital of Hainan Medical University Haikou Hainan China; ^3^ Department of Dermatology Haikou Affiliated Hospital of Central South University Xiangya School of Medicine Haikou Hainan China

**Keywords:** cerebral ischemia‐reperfusion injury, ETV4, ferroptosis, GPX4, YBX1

## Abstract

**Background:**

Cerebral ischemia‐reperfusion injury (CIRI) is a critical pathological process following ischemic stroke, with ferroptosis being increasingly recognized as a key contributor to neuronal damage. However, the regulatory mechanisms, particularly the role of specific transcription factors like E26 transformation‐specific variant 4 (ETV4), remain poorly understood. This study aimed to investigate the function and underlying mechanism of ETV4 in neuronal ferroptosis during CIRI.

**Methods:**

In vitro, SH‐SY5Y cells subjected to oxygen‐glucose deprivation/reoxygenation (OGD/R) were used to model CIRI. Cell viability and ferroptosis markers (Fe^2^^+^, malondialdehyde (MDA), glutathione (GSH), superoxide dismutase (SOD), reactive oxygen species (ROS)) were assessed. Molecular expressions were measured by Real‐time Quantitative PCR (RT‐qPCR) and/or western blot. In vivo, a rat model of middle cerebral artery occlusion/reperfusion (MCAO/R) was established, and brain injury was evaluated via 2% solution of 2,3,5‐triphenyl tetrazolium chloride (TTC), hematoxylin‐eosin (HE), and TdT‐mediated dUTP nick‐end labeling (TUNEL) staining. Chromatin immunoprecipitation (ChIP), dual‐luciferase reporter, RNA pull‐down, RNA immunoprecipitation (RIP), and actinomycin D assays were employed to validate the molecular interactions within the ETV4/Y‐box binding protein 1 (YBX1)/glutathione peroxidase‐4 (GPX4) axis.

**Results:**

ETV4 was significantly down regulated in both the MCAO/R model and OGD/R‐treated cells. Overexpression of ETV4 markedly attenuated OGD/R‐induced oxidative stress, ferroptosis in vitro, and ameliorated brain injury in vivo. Mechanistically, ETV4 transcriptionally activated YBX1 by directly binding to its promoter. YBX1, in turn, stabilized GPX4 mRNA, which was modified by NSUN2‐mediated methylation. Crucially, the protective effects of ETV4 in vitro were abolished upon YBX1 or GPX4 knockdown.

**Conclusion:**

Our findings demonstrate that ETV4 transcriptionally up regulates YBX1 to stabilize GPX4 mRNA in an NSUN2–m5Cmethylation dependent manner, thus suppressing neuronal ferroptosis. This reveals a novel ETV4/YBX1/GPX4 axis as a potential therapeutic target for CIRI.

## Graphical abstract

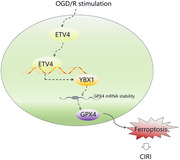

**Abbreviations**
CCK‐8: cell Counting Kit‐8
cDNA: complementary DNA
ChIP: chromatin immunoprecipitation
CIRI: cerebral ischemia‐reperfusion injury
ETS: E26 transformation‐specific
ETV4: E26 transformation‐specific variant 4
FBS: fetal bovine serum
GPX4: glutathione peroxidase‐4
GSH: glutathione
HE: hematoxylin‐eosin
I/R: ischemia/reperfusion
LV: lentiviral
m5C: 5‐methyladenosine
MCAO: middle cerebral artery occlusion
MDA: malondialdehyde
NEAAs: non‐essential amino acids
OGD/R: oxygen and glucose deprivation/reoxygenation
PGIs: post‐synaptic GABAergic interneurons
PI: propidium iodide
RIP: RNA immunoprecipitation
ROS: reactive oxygen species
SOD: superoxide dismutase
TdT: terminal deoxynucleotidyl transferase
TTC: 2% solution of 2,3,5‐triphenyl tetrazolium chloride
TUNEL: TdT‐mediated dUTP nick‐end Labeling
VGLUT3: vesicular glutamate transporter‐3
YBX1: Y‐box binding protein 1

## 1 Introduction

Cerebral ischemia‐reperfusion injury (CIRI) is a major therapeutic paradox that arises when blood flow is restored to ischemic brain tissue, paradoxically amplifying the primary insult and worsening clinical outcomes (Yang et al. 2022). It is a major contributor to morbidity and mortality in patients suffering from ischemic stroke, a leading cause of disability and death worldwide. Current clinical management focuses on timely reperfusion strategies, primarily through thrombolytic therapy and mechanical thrombectomy. However, the success of these treatments is often limited by narrow therapeutic time windows, risks of hemorrhagic complications, and the paradoxical exacerbation of injury upon reperfusion (Zhang et al. 2024). Pathologically, CIRI is orchestrated by a highly interconnected pathological cascade that begins with mitochondrial energy metabolism disorder and excitotoxic glutamate accumulation, followed by oxidative stress, Ca^2^^+^ overload, blood‐brain barrier disruption, and sterile neuro‐inflammation (Kapanova et al. 2022). Within this network, programmed neuronal death emerges as the final, irreversible step that dictates the extent of tissue loss and neurological deficit (Zhang et al. 2022). Therefore, elucidating the precise molecular mechanisms underlying neuronal death is not only crucial for deepening our understanding of CIRI pathophysiology but also of great scientific significance.

Ferroptosis is an iron‐dependent, lipid‐peroxidation‐driven modality of programmed cell death that is morphologically, biochemically, and genetically distinct from apoptosis, necroptosis, and pyroptosis (Dixon et al. 2012). It is characterized by lethal accumulation of polyunsaturated fatty acid (PUFA)‐derived phospholipid hydro peroxides in plasma membranes, a process that requires free Fe^2^^+^‐catalyzed Fenton chemistry and is typically preceded by mitochondrial shrinkage, increased membrane density, and disappearance of cristae (Tang et al. 2021). Central to this cascade is glutathione peroxidase‐4 (GPX4), the only enzyme capable of reducing phospholipid hydroperoxides to harmless alcohols using GSH as a cofactor; hence, GPX4 depletion or inactivation is both necessary and sufficient to trigger ferroptosis (Yang et al. 2014). Over the past decade, ferroptosis has emerged as a major pathogenic mechanism in CIRI (Liu et al. 2024). A large number of reports have shown that targeting neuronal ferroptosis exerts neuroprotective effects against CIRI (Li et al. 2022; Wang et al. 2023). Pharmacological or genetic blockade of ferroptosis‐via iron chelators, lipophilic antioxidants, or neuron‐specific GPX4 over‐expression‐markedly reduces infarct volume and neurological deficits in rodent stroke models (Hu et al. 2024). Despite its recognition as a promising therapeutic target, the upstream molecular network that precisely governs ferroptosis in the context of CIRI remains inadequately elucidated.

The E26 transformation‐specific (ETS) family of transcription factors is characterized by a highly conserved DNA‐binding domain that recognizes purine‐rich motifs in gene promoters or enhancers, thereby orchestrating context‐dependent transcriptional programs (Wang et al. 2023). Within this family, ETS variant 4 (ETV4) is well‐documented for its role in embryonic neuronal development, including neuronal specification and axonal guidance (Qi et al. 2023; Gadiraju et al. 2024). Previously, our previous work revealed that ETV4 contributes to the protective effect of BMSC‐exosomal lncRNA KLF3‐AS1 against CIRI by activating Sirt1‐dependent autophagy (Xu et al. 2023). Moreover, ETV4 transcriptionally up‐regulates vesicular glutamate transporter‐3 (VGLUT3) in hippocampal neurons, thereby potentiating VGLUT3‐positive glutamatergic synapses onto post‐synaptic GABAergic interneurons (PGIs) and rescuing spatial memory deficits in APP/PS1 Alzheimer's disease mice (He et al. 2022), suggesting a possible involvement of ETV4 in the maintenance of neural function. Nevertheless, the aberrant expression of ETV4 in CIRI and its precise function in neuronal damage remain largely unexplored.

Emerging evidence links ETV4 to the regulation of ferroptosis, a key pathological process in CIRI. As reports, ETV4 can transcriptionally activate key ferroptosis regulators such as SLC7A11 or GPX4, thereby modulating cellular sensitivity to ferroptosis (He et al. 2024; Wang et al. 2021). Thus, we aimed to probe whether ETV4 can influence CIRI via regulating neuronal ferroptosis. In this study, we demonstrate that ETV4 attenuates CIRI by suppressing neuronal ferroptosis through transcriptional up regulation of Y‐box binding protein 1 (YBX1), which in turn stabilizes GPX4 mRNA. Our findings reveal a novel ETV4/YBX1/GPX4 axis, providing fresh insights and potential therapeutic targets for CIRI.

## 2 Materials and Methods

### 2.1 Cell Culture and Treatment

Human neuroblastoma SH‐SY5Y cell line was sourced from the American Type Culture Collection (ATCC, Manassas, VA). These cells were grown in Dulbecco's modified Eagle's medium F‐12 (DMEM/F‐12; Gibco, Grand Island, NY, USA) supplemented with 10% fetal bovine serum (FBS; Gibco) and 1% non‐essential amino acids (NEAAs; Gibco) at 37°C in a 5% CO_2_ environment. For the oxygen‐glucose deprivation/reoxygenation (OGD/R) model, SH‐SY5Y cells were cultured in glucose‐free DMEM/F‐12 and placed in a hypoxic chamber (1% O_2_, 94% N_2_, 5% CO_2_) for 4 h. Subsequently, the medium was replaced with complete DMEM/F‐12, and cells were transferred back to a normoxic environment (21% O_2_, 5% CO_2_) for 3, 6, 12, or 24 h to simulate reperfusion.

### 2.2 Cell Transfection

The synthetic shRNA targeting NSUN2 (sh‐NSUN2), YBX1 (sh‐YBX1), and ETV4(sh‐ETV4#1, sh‐ETV4#2), overexpression plasmids for ETV4 (pcDNA3.1‐ETV4), YBX1 (pcDNA3.1‐YBX1), NSUN2 (pcDNA3.1‐NSUN2), and GPX4 (pcDNA3.1‐GPX4), and their respective negative controls (shNC, pcDNA3.1) were obtained from GenePharma (Shanghai, China). For cell transfection, the above plasmids or shRNAs were transfected into cells utilizing Lipofectamine 3000 (Invitrogen, Carlsbad, CA, USA) following the manufacturer's instruction. The transfection efficiency was evaluated at 48 h post‐transfection.

### 2.3 Total RNA Extraction and Real‐Time Quantitative PCR (RT‐qPCR)

Total mRNA was extracted utilizing TRIzol reagent (Invitrogen). RNA was converted into complementary DNA (cDNA) employing PrimeScriptTM RT Reagent Kit (TaKaRa, Tokyo, Japan). Real‐time PCR was performed utilizing SYBR Green qPCR (Applied Biosystems, Carlsbad, CA, USA) on an ABI Prism 7900 HT Sequence Detection System (Thermo Fisher Scientific, Waltham, MA, USA). The primers for human ETV4 F: 5′‐GAAAAACAAGTCGGTGCGCT‐3′, R: 5′‐TTGCTGCTGAAGGTGTAGGG‐3′; rat ETV4 F: 5′‐TGGCCACAAAGGCGGATATT‐3′, R: 5′‐CACTGTCTTTCCCGCAGACT‐3′; human YBX1 F: 5′‐AAGTGATGGAGGGTGCTGAC‐3′, R: 5′‐TGACCTTGGGTCTCATCTCC‐3′; human GPX4 F: 5′‐TCACCAAGTTTGGACACCGT‐3′, R: 5′‐ATAGTGGGGCAGGTCCTTCT‐3′; human NSUN2 F: 5′‐GCTACCCCGAGATCGTCAAG ‐3′, R: 5′‐TCAGGATACCTTTTGTAACCAGT‐3′. Human GAPDH F: 5′‐CTGACTTCAACAGCGACACC‐3′, R: 5′‐GTGGTCCAGGGGTCTTACTC‐3′; rat GAPDH F: 5′‐GCAAGTTCAACGGCACAG‐3′, R: 5′‐GCCAGTAGACTCCACGACAT‐3′. GAPDH is used as an endogenous control. Expression levels of mRNA were quantified and calculated by the 2^−∆∆Ct^ method.

### 2.4 Western Blot

Total proteins of brain tissues or cells were extracted utilizing RIPA lysis buffer (Beyotime, Shanghai, China). Protein concentrations were quantified by a BCA kit (Beyotime). Protein samples underwent separation through 12% SDS‐PAGE and were subsequently transferred to PVDF membranes (Millipore, Billerica, MA, USA). After blocking in 5% BSA (Sigma, St. Louis, MO, USA), membranes were incubated overnight at 4°C with primary antibodies: ETV4 (PA5‐99226, 1:1000, Invitrogen), YBX1 (PA5‐83493, 1:1000, Invitrogen), and GPX4 (MA5‐32827, 1:1000, Invitrogen). The following day, membranes were washed with TBST and then incubated with HRP‐conjugated secondary antibodies (#7074, 1:1,000, Cell Signaling Technology, Danvers, MA, USA). Bands were performed with an ECL Chemiluminescence Kit (Beyotime) and visualized by ImageJ software (NIH, Bethesda, MD, USA). β‐actin served as an internal control.

### 2.5 Cell Counting Kit‐8 (CCK‐8) Assay

CCK‐8 (Beyotime, Shanghai, China) was employed to assess cell viability. Briefly, SH‐SY5Y cells were seeded into 96‐well plates at a density of 5 × 10^3^ cells per well and cultured for 24 h under standard conditions (37°C, 5% CO_2_) to allow for adherence. After the respective treatments, 10 µL of CCK‐8 solution was introduced to each well and incubated for another 2 h. The optical density (OD) at a wavelength of 450 nm was then measured using a microplate reader (Thermo Fisher Scientific, USA).

### 2.6 Live/Dead Cell Staining

The Calcein AM/PI Staining Kit (Beyotime) was used to analyze the cell death rate of SH‐SY5Y cells, according to the manufacturer's protocol. Briefly, after the respective treatments, SH‐SY5Y cells were harvested and stained with 2 µM Calcein‐AM and 15 µg/mL PI at 37°C for 30 min in the dark. Afterwards, cells were washed and immediately captured using a flow cytometer (BD FACSCalibur, BD Biosciences, San Jose, CA, USA).

### 2.7 Determination of Iron Ion Content

Cellular Fe^2^^+^ levels were quantified using a commercial Iron Assay Kit (Abcam). Following the protocol, SH‐SY5Y cell lysates from each group were prepared, and the supernatant was reacted with the working reagent at 37°C for 60 min in the dark. Absorbance was then measured at 593 nm using a microplate reader (Thermo Fisher Scientific).

### 2.8 Detection of Oxidative Stress Factors

Concentrations of malondialdehyde (MDA), glutathione (GSH), and superoxide dismutase (SOD) were evaluated in homogenized brain tissues and cell lysates using the respective Assay Kits (Beyotime). The assay procedures followed the manufacturer's guidelines, and the final absorbance was detected at 450 nm with a microplate reader (Thermo Fisher Scientific).

### 2.9 Intracellular Reactive Oxygen Species (ROS) Detection

The ROS Assay Kit, purchased from Beyotime, was used to examine the changes in ROS levels. Cells were incubated with serum‐free medium containing 10 µM 2',7'‐dichlorodihydrofluorescein diacetate (DCFH‐DA) for 20 min at 37°C. After incubation, the cells were washed three times and immediately captured using a fluorescence microscope (Olympus).

### 2.10 Establishment of the Middle Cerebral Artery Occlusion and Reperfusion (MCAO/R) Model

Male Sprague‐Dawley (SD) rats, weighing between 220–250 g and aged 7–8 weeks, were obtained from the Experimental Animal Center at Guangzhou Huateng Biopharmaceutical Technology Co., Ltd. Animals were housed in a room with a 12 h light/dark cycle at 22°C, with access to food and water ad libitum. Before commencement of the experiment, rats were allowed unrestricted access to food and water and were acclimated for a period of 1 week. Rats were randomly divided into four groups (*n* = 5/group): sham, MCAO/R‐12 h, MCAO/R‐24 h, MCAO/R‐48, MCAO/R‐24h + LV‐NC, and MCAO/R‐24 h + LV‐ETV4. To overexpress ETV4, rats in the I/R + LV‐ETV4 group received an intracerebroventricular injection of lentivirus carrying the ETV4 gene (1 × 10^8^ TU/mL, GenePharma) 72 h prior to MCAO/R induction, while the I/R + LV–NC group was injected with a control lentivirus. Briefly, under anesthesia induced by sodium pentobarbital (65 mg/kg, i.p.), rats were positioned in a stereotaxic apparatus. Using coordinates relative to bregma (anteroposterior: −0.8 mm; mediolateral: ±1.4 mm; dorsoventral: −3.5 mm) (Shen et al. 2025), the lentivirus was infused into the left lateral ventricle at a rate of 0.5 µL/min. The cannula was left in place for 5 min post‐infusion before being slowly withdrawn. Next, the MCAO/R model was established as previously described (Shen et al. 2025). Briefly, after being anesthetized, a midline cervical incision was made to expose the left common carotid artery, external carotid artery, and internal carotid artery. A silicone‐coated monofilament suture was then inserted into the internal carotid artery to occlude the origin of the middle cerebral artery for 2 h. The suture was withdrawn to initiate reperfusion for 12 h, 24 h, or 48 h, respectively. Sham‐operated rats underwent the same surgical procedure except for artery occlusion. After reperfusion, rats were euthanized, and brain tissues were collected for subsequent analysis. All experimental procedures were approved by the Animal Ethics Committee of Haikou Affiliated Hospital of Central South University Xiangya School of Medicine. The procedures were performed in accordance with the ARRIVE guidelines.

### 2.11 Infarct Volume Measurement

Following 24 h of reperfusion, brains were rapidly harvested and sectioned coronally into 2‐mm‐thick slices. The brain slices were incubated in a 2% solution of 2,3,5‐triphenyltetrazolium chloride (TTC; Thermo Fisher Scientific) at 37°C for 30 min in the dark, followed by overnight fixation in 4% paraformaldehyde. The stained sections were photographed, and infarct areas were quantified using ImageJ software (NIH). The percentage infarct volume was calculated as follows: infarct volume (%) = [(volume of contralateral hemisphere) – (volume of non‐infarcted ipsilateral hemisphere)]/(volume of contralateral hemisphere) × 100.

### 2.12 Hematoxylin‐Eosin (HE) Staining

Brain tissues were fixed in 4% paraformaldehyde overnight, embedded in paraffin, and sectioned at a thickness of 5 µm. Following deparaffinization in xylene and rehydration through a graded ethanol series, the sections were stained with hematoxylin for 5 min and then rinsed with distilled water. Subsequently, the sections were counterstained with eosin solution for 2 min and rinsed again. After dehydration and clearing, the sections were mounted with neutral resin. Histopathological images were acquired using a light microscope (Olympus).

### 2.13 TdT‐Mediated dUTP Nick‐End Labeling (TUNEL) Staining

Paraffin‐embedded brain Sections (5 µm) were deparaffinized and rehydrated. The TUNEL assay was performed using a commercial kit (Beyotime) as per the manufacturer's protocol. Briefly, after pretreatment with proteinase K, sections were incubated with an equilibration buffer, followed by a TdT‐dUTP reaction mixture at 37°C for 1 h in the dark. The reaction was stopped, and nuclei were counterstained with DAPI. TUNEL‐positive cells (green) and DAPI‐stained nuclei (blue) were visualized and counted under a fluorescence microscope (Olympus).

### 2.14 Chromatin Immunoprecipitation (ChIP)

SH‐SY5Y cells were cross‐linked with 1% formaldehyde for 10 min at room temperature, and a 0.125 M final concentration of glycine was added to quench the cross‐linking. Then, cells were harvested and lysed using ice‐cold lysis buffer. Then, chromatin was sheared by sonication to an average length of 200–500 bp. The sonicated lysates were next incubated overnight at 4°C with rotation with anti‐ETV4 antibody (10684‐1‐AP, Proteintech, Wuhan, Hubei, China) or an equivalent normal IgG (Abcam, ab171870) pre‐bound to protein A/G magnetic beads. The bound complexes were eluted, and cross‐links were reversed by incubation at 65°C overnight with proteinase K. The immunoprecipitated DNA was purified and analyzed by RT‐qPCR. The primers used for ChIP–qPCR were forward primer: CGTGGCTGTTGCAGGAATAA and reverse primer: GGTTCAGCATGTTTAGGGCG, amplifying a 205 bp product located from −836–−631 upstream of the YBX1 transcription start site (chr1: 42,681,254–42,681,049).

### 2.15 Dual Luciferase Reporter Assay

The promoter fragment of YBX1 (chromosome 1:42680418–42682468) was cloned and inserted into pGL3 Control Vector (Promega, Madison, WI, USA) to generate reporter vectors YBX1. Then, the recombinant luciferase reporter vectors were co‐transfected with pcDNA3, 1‐ETV4, or pcDNA3.1 into SH‐SY5Y cells using Lipofectamine 2000 (Invitrogen). After 48 h, luciferase relative activity was determined utilizing the Dual‐Luciferase Reporter Assay System (Promega).

### 2.16 RNA Immunoprecipitation (RIP)

The RIP experiment was performed using the Magna RIP RNA‐Binding Protein Immunoprecipitation Kit (Millipore). Following formaldehyde cross‐linking, cells were collected, suspended, and centrifuged. Subsequently, cells were resuspended in RIP buffer containing protease and RNase inhibitors. They were then incubated overnight with magnetic beads conjugated with anti‐YBX1 (PA5‐83493, 1:1000, Invitrogen), anti‐NSUN2 (MA5‐56501, 1:1000, Invitrogen), anti‐m5C (ab214727, Abcam), or IgG (ab172730, Abcam) with rotation. Following elution, isolated mRNA was analyzed using RT‐qPCR.

### 2.17 RNA Pull Down

Biotin‐labeled sense and antisense RNA probes targeting GPX4 were synthesized by Roche (Basel, Switzerland). The probes were incubated with streptavidin magnetic beads to form probe‐bead complexes. Cell lysates were then added to the complexes and incubated to allow RNA‐protein binding. After washing, the proteins bound to the biotinylated RNA were eluted and analyzed by western blot to detect the interaction with YBX1 and NSUN2.

### 2.18 mRNA Stability Test

To assess the effect of YBX1 on GPX4 mRNA stability, SH‐SY5Y cells were transfected with shRNA against YBX1, YBX1 overexpression vector (pcDNA3.1‐YBX1), as well as their negative controls (shNC, pcDNA3.1) for 48 h. Following confirmation of successful YBX1 overexpression or knockdown, the transfected cells were exposed to Actinomycin D (5 µg/mL, Sigma) for 0, 2, 4, 6, and 8 h. Then, cells were harvested and the remaining RNA was extracted and the remaining GPX4 mRNA was examined by RT‐qPCR analysis.

### 2.19 Statistical Analysis

Statistical analyses were performed utilizing GraphPad Prism 8.0 software (San Diego, CA, USA). All values are presented as mean ± standard deviation (SD). Each experiment was performed in triplicate. Comparisons between two groups were conducted using Student's t‐test. Differences among multiple groups were analyzed by one‐way analysis of variance (ANOVA). *P* < 0.05 was considered significantly differences.

## 3 Results

### 3.1 ETV4 was Decreased in Brain Tissues of MCAO/R Rats and OGD/R‐Induced SH‐SY5Y Cells

**FIGURE 1:**
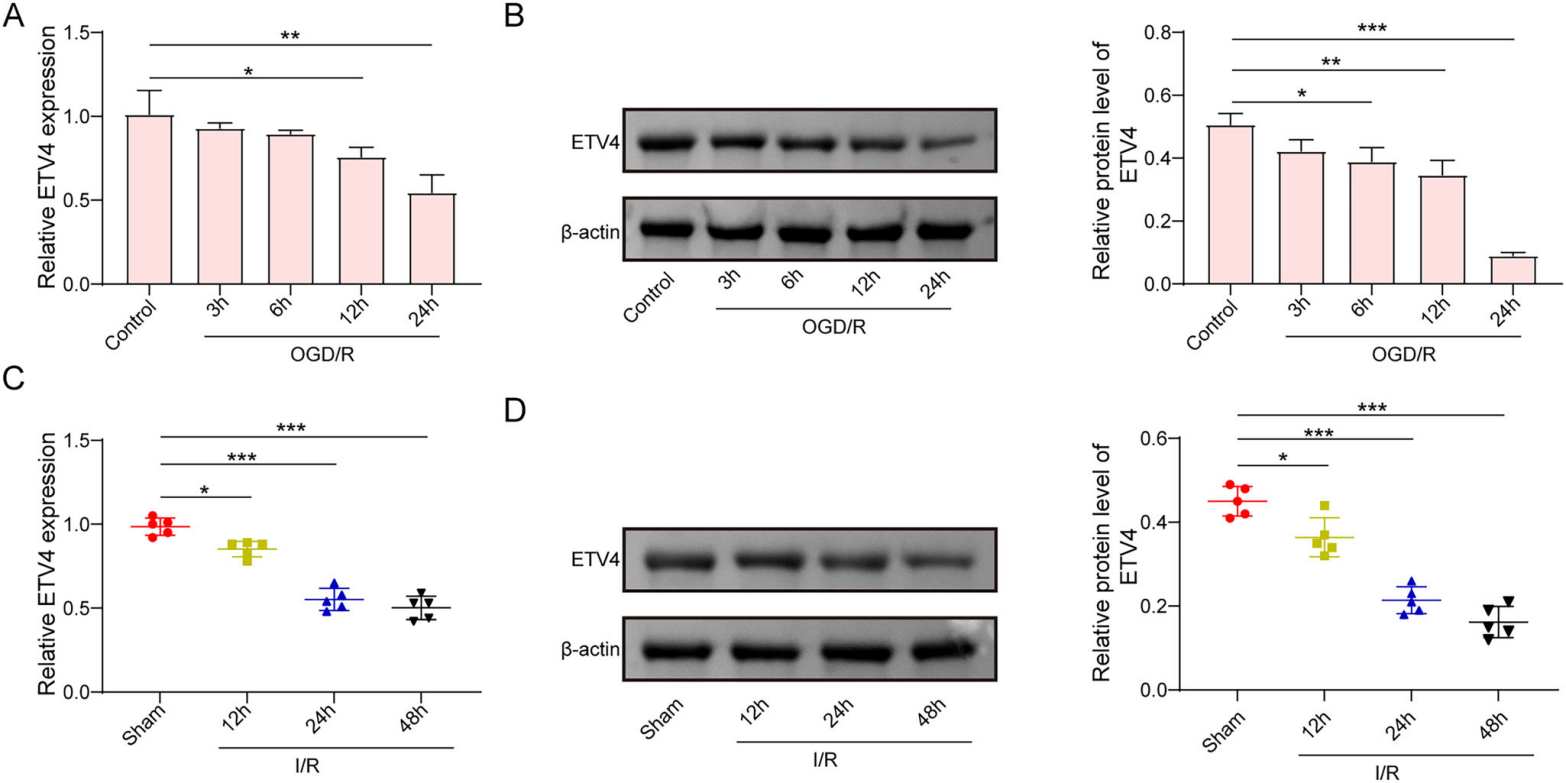
ETV4 was decreased in brain tissues of MCAO/R rats and OGD/R‐induced SH‐SY5Y cells. (A, B) SH‐SY5Y neuroblastoma cells were subjected to OGD/R for 3 h, 6 h, 12 h, or 24 h. ETV4 mRNA and protein levels were measured using RT‐qPCR and western blot. (C, D) SD rats underwent 2 h MCAO followed by reperfusion for 12, 24, or 48 h. ETV4 mRNA and protein levels in brain tissues were examined utilizing RT‐qPCR and western blot. *n* = 5 rats/group. Data are presented as mean ± SD. *P < 0.05, **P < 0.01, and ***P < 0.001.

The dynamic changes of ETV4 expression in MCAO/R rats and OGD/R‐triggered SH‐SY5Y cells were investigated. As shown in Figures 1A and 1B, ETV4 mRNA and protein levels were declined in a time‐dependent manner following OGD/R treatment. Moreover, it was also found that ETV4 was down regulated in brain tissues of MCAO/R rats compared to the sham group, with a more pronounced reduction observed at longer reperfusion durations (Figures 1C and 1D). However, no statistically significant difference in ETV4 expression was found between the 24 h and 48 h reperfusion time points (Figures 1C and 1D). Collectively, these data demonstrate that ETV4 suppression was closely associated with CIRI.

### 3.2 ETV4 Modulated OGD/R‐Stimulated Oxidative Stress and Ferroptosis in SH‐SY5Y Cells

**FIGURE 2:**
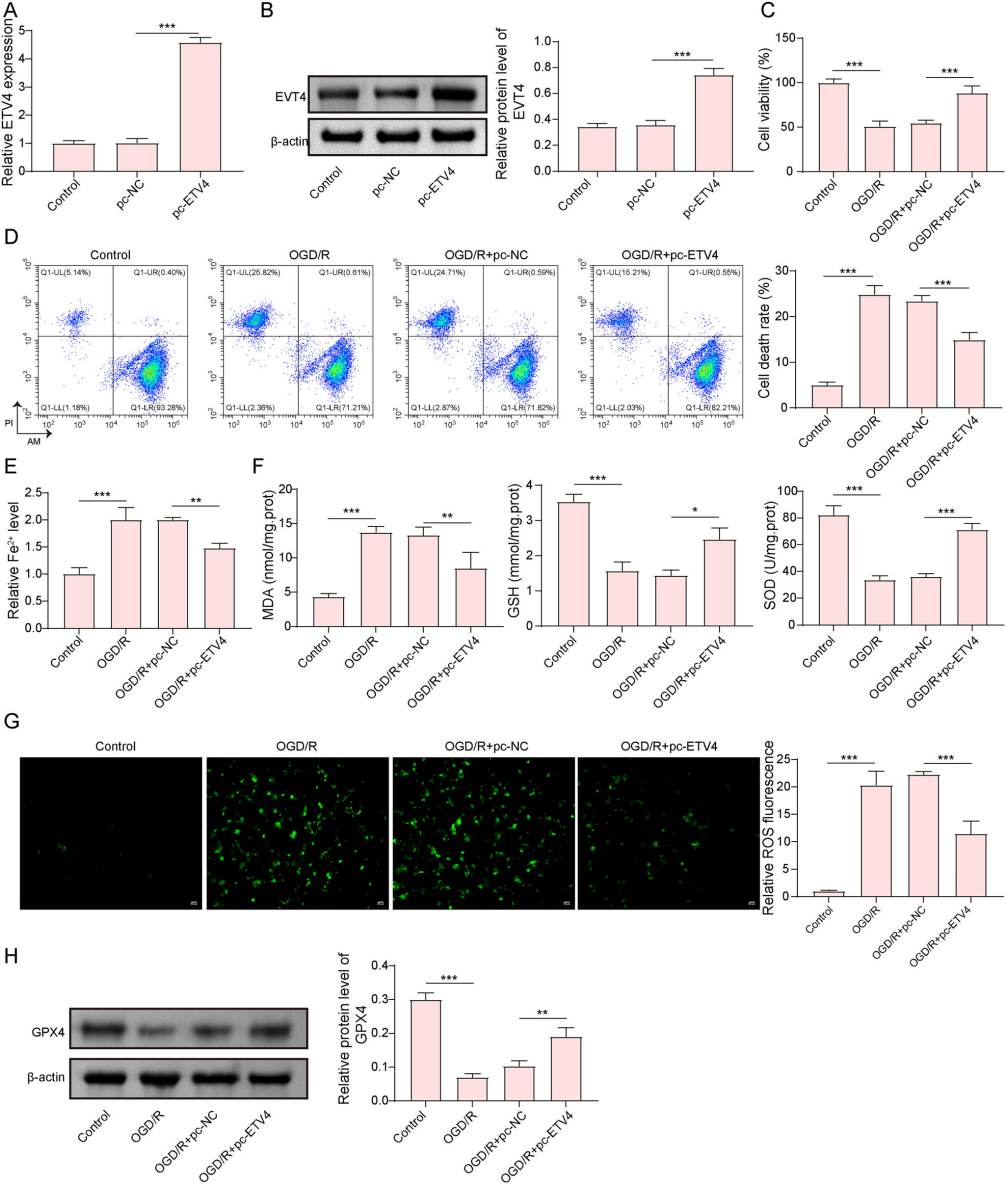
ETV4 modulated OGD/R‐stimulated oxidative stress and ferroptosis in SH‐SY5Y cells. (A) RT‐qPCR validation of ETV4 mRNA in SH‐SY5Y cells transfected with empty vector (pcDNA3.1) or ETV4‐overexpressing plasmid (pcDNA3.1‐ETV4). SH‐SY5Y cells were transfected with pcDNA3.1 or pcDNA3.1‐ETV4 for 48 h, followed by exposure to OGD/R; (B) western blot validation of ETV4 protein expression in SH‐SY5Y cells transfected with pcDNA3.1 or pcDNA3.1‐ETV4. SH‐SY5Y cells were transfected with pcDNA3.1 or pcDNA3.1‐ETV4 for 48 h, followed by exposure to OGD/R; (C) CCK‐8 assay for cell viability; (D) Calcein‐AM/PI staining for determining cell death rate; (E) ELISA quantification of intracellular Fe^2^^+^ accumulation; (F) ELISA measurement of MDA, GSH, and SOD levels. (G) DCFH‐DA assessment of intracellular ROS level; and (H) western blot was used to test GPX4 expression. Data are mean ± SD from three independent experiments. *P < 0.05, **P < 0.01, and ***P < 0.001.

To determine whether ETV4 contributes to neuronal ferroptosis in vitro, we overexpressed ETV4 in SH‐SY5Y cells using a pcDNA3.1‐ETV4 plasmid. Transfection efficiency was confirmed by RT‐qPCR, which showed a significant increase in ETV4 mRNA levels compared to the empty vector control (Figure 2A). In addition, western blot analysis further confirmed a marked increase in ETV4 protein expression in cells transfected with pcDNA3.1‐ETV4 compared with the empty vector control (Figure 2B). Under OGD/R conditions, ETV4 overexpression significantly improved cell viability (Figure 2C) and reduced the cell death rate (Figure 2D). Furthermore, ETV4 up regulation reversed the OGD/R‐induced increases in Fe^2^^+^ and MDA levels, as well as the decreases in GSH and SOD activity (Figure 2E and 2F). ETV4 overexpression also markedly attenuated intracellular ROS accumulation in OGD/R‐induced SH‐SY5Y cells (Figure ). Western blot assay also presented that GPX4 protein was down regulated by OGD/R, and ETV4 overexpression rescued this downward trend (Figure 2H).

To further validate the role of ETV4 in neuronal ferroptosis, ETV4 was knocked down in SH‐SY5Y cells using two independent short hairpin RNAs (sh‐ETV4#1 and sh‐ETV4#2). RT‐qPCR and western blot analyses confirmed that both shRNAs significantly reduced ETV4 mRNA and protein expression compared with the shNC group (Supplementary Figure 1A and 1B). Under OGD/R conditions, ETV4 knockdown markedly decreased cell viability (Supplementary Figure 1C) and increased the cell death rate relative to the shNC group (Supplementary Figure 1D). Additionally, silencing of ETV4 further enhanced OGD/R‐induced intracellular Fe^2^^+^ accumulation, elevated MDA levels, and exacerbated the reductions in GSH content and SOD activity (Supplementary Figure 1E and 1F). Consistently, ETV4 deficiency led to a pronounced increase in ROS production in OGD/R‐exposed cells (Supplementary Figure 1G). Western blot analysis revealed that GPX4 protein levels, which were reduced upon OGD/R treatment, were further diminished following ETV4 knockdown (Supplementary Figure 1H). Collectively, these results indicate that ETV4 overexpression antagonizes, whereas ETV4 depletion aggravates, OGD/R‐evoked oxidative stress and ferroptosis in SH‐SY5Y cells, supporting a protective role of ETV4 against neuronal ferroptosis.

### 3.3 Overexpression of ETV4 Alleviated I/R‐Induced Neuronal Ferroptosis in Vivo

**FIGURE 3:**
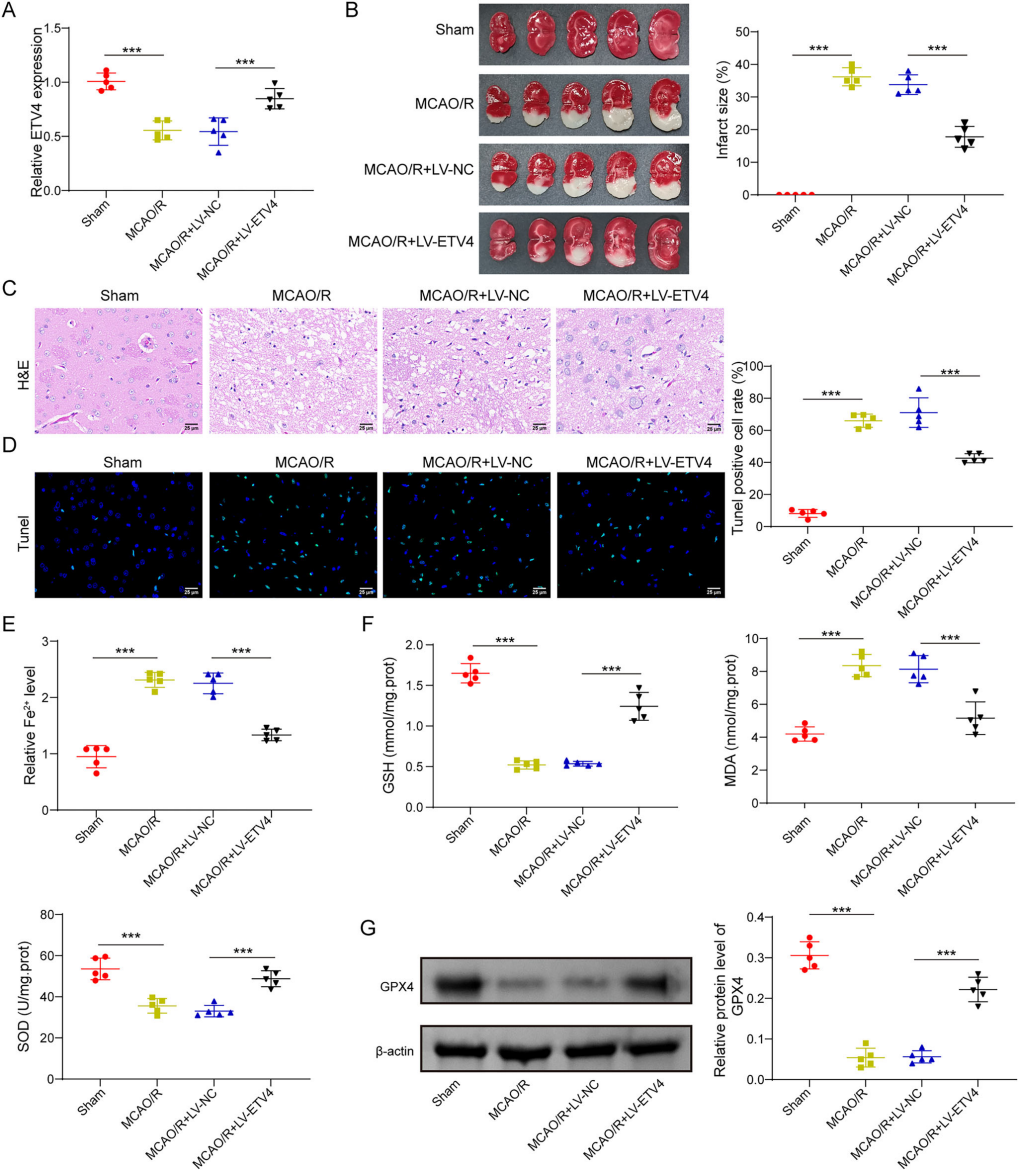
Overexpression of ETV4 alleviated I/R‐induced neuronal ferroptosis in vivo. SD rats received an injection of lentivirus carrying ETV4 (LV‐ETV4) or empty vector (LV‐NC) 72 h prior to MCAO/R. (A) The mRNA expression level of ETV4 in the brain tissue was measured by RT‐qPCR; (B) representative TTC‐stained coronal sections and quantitative infarct volume (white areas); (C) histopathological changes in brain tissue were observed by HE staining; (D) TUNEL staining detected neuronal death; (E) ELISA quantification of intracellular Fe^2^^+^ accumulation; (F) ELISA measurement of MDA, GSH, and SOD levels; and (G) western blot was used to test GPX4 expression. Data are mean ± SD from three independent experiments. *P < 0.05, **P < 0.01, and ***P < 0.001.

To further validate the protective role of ETV4 in vivo, we constructed a rat model of MCAO/R, and overexpressed ETV4 via intracerebroventricular injection of a lentiviral vector (LV‐ETV4). First, qRT‐PCR analysis confirmed the successful overexpression of ETV4 in the brain tissues of the MCAO/R + LV‐ETV4 group compared with the MCAO/R and MCAO/R + LV‐NC groups (Figure 3A). TTC staining revealed a large infarct area in the MCAO/R group, which was significantly reduced by LV‐ETV4 treatment (Figure 3B). Histological examination by HE staining showed severe neuronal damage, including disordered arrangement, shrunken cell bodies, and nuclear pyknosis in the MCAO/R group, and these pathological changes were markedly ameliorated in the MCAO/R + LV‐ETV4 group (Figure 3C). Furthermore, TUNEL staining demonstrated a substantial increase in apoptotic neurons following MCAO/R injury, which was effectively suppressed by ETV4 overexpression (Figure 3D). In addition, the Fe^2^^+^ and MDA levels were up regulated in brain tissues of MCAO/R rats, and GSH and SOD levels were down regulated, while these effects were eliminated by ETV4 overexpression (Figures 3E and 3F). Finally, the protein level of GPX4 was drastically decreased in the MCAO/R group, while ETV4 overexpression restored its expression (Figure 3G). These in vivo data corroborate the cell‐based findings and demonstrate that overexpression of ETV4 curbs iron accumulation and neuronal death.

### 3.4 ETV4 Transcriptional Activated YBX1 Expression

**FIGURE 4:**
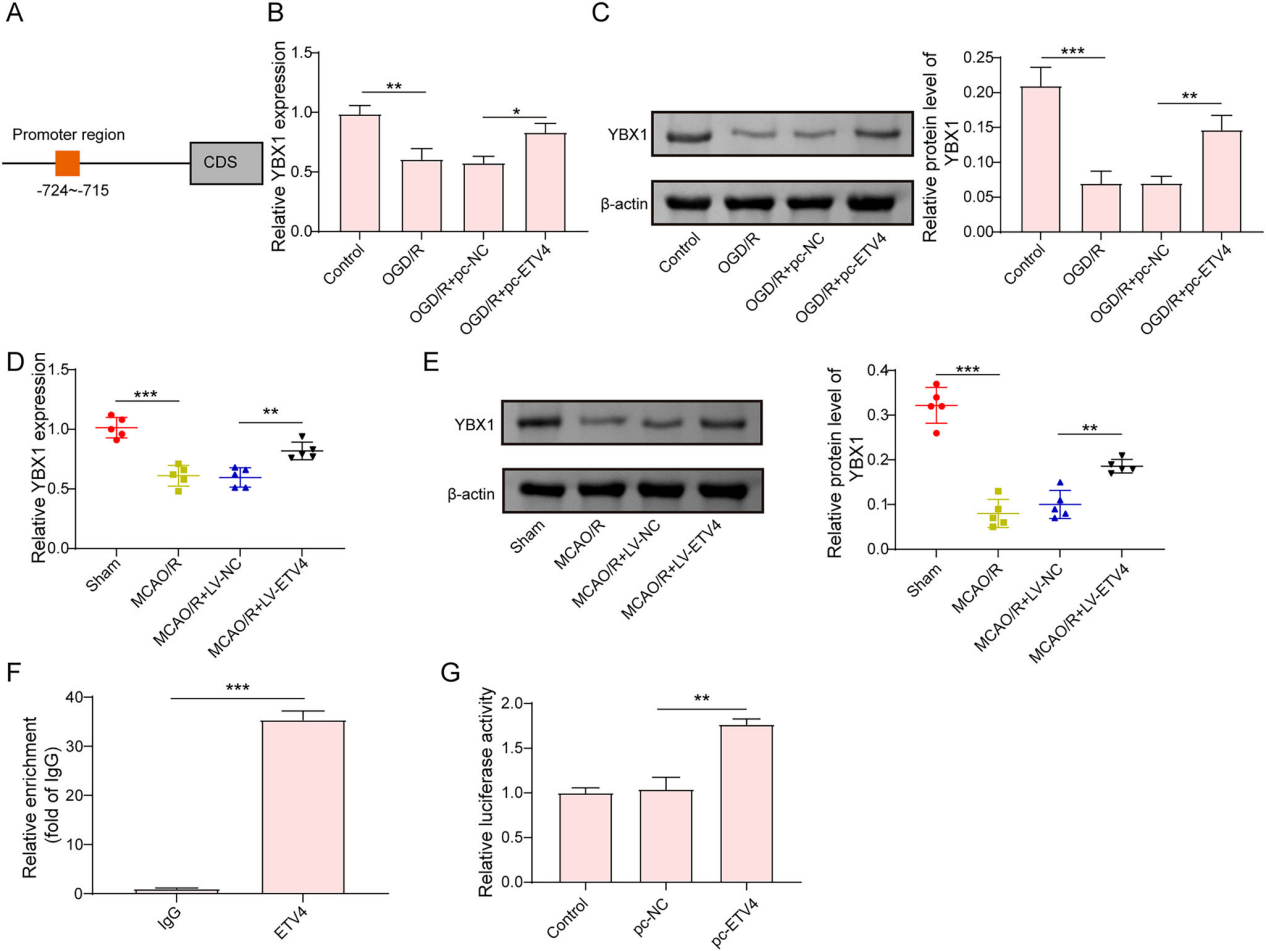
ETV4 transcriptional activated YBX1 expression. (A) JASPAR database predicted the binding site of ETV4 in YBX1 promoter region. SH‐SY5Y cells were transfected with pcDNA3.1 or pcDNA3.1‐ETV4 for 48 h, followed by exposure to OGD/R. (B, C) The levels of YBX1 were detected by RT‐qPCR and western blot, respectively. (D, E) rats were subjected to MCAO/R after intracerebroventricular injection of LV‐NC or LV‐ETV4. The mRNA and protein levels of YBX1 in brain tissues were measured by RT‐qPCR and western blot; (F) the binding relationship between ETV4 and YBX1 promoter was detected by ChIP assay; and (G) the effect of ETV4 on transcriptional activity of YBX1 was detected by dual‐luciferase reporter assay. Data are mean ± SD from three independent experiments. *P < 0.05, **P < 0.01, and ***P < 0.001.

To explore the downstream mechanisms by which ETV4 exerts its protective effects, we investigated its potential transcriptional targets. Bioinformatics analysis using the JASPAR database predicted that ETV4 contains a high‐confidence binding site within the promoter region of YBX1 (Figure 4A). In OGD/R‐injured SH‐SY5Y cells, the mRNA and protein levels of YBX1 were significantly decreased, whereas, this effect was effectively rescued by ETV4 overexpression (Figures 4B and 4C). Consistent with the cellular results, YBX1 mRNA and protein were decreased in brain tissues after MCAO/R treatment compared to sham group, while this decrease was reversed by lentivirus‐mediated overexpression of ETV4 (Figures 4D and 4E). In addition, ChIP assay demonstrated a significant enrichment of the YBX1 promoter region in the anti‐ETV4 antibody compared with the IgG group (Figure 4F). Additionally, a dual‐luciferase reporter assay demonstrated that ETV4 overexpression significantly enhanced the transcriptional activity of the YBX1 promoter (Figure 4G). Therefore, these results demonstrate that ETV4 transcriptionally activates YBX1 by binding to its promoter.

### 3.5 YBX1 Maintained the mRNA Stability of GPX4

**FIGURE 5:**
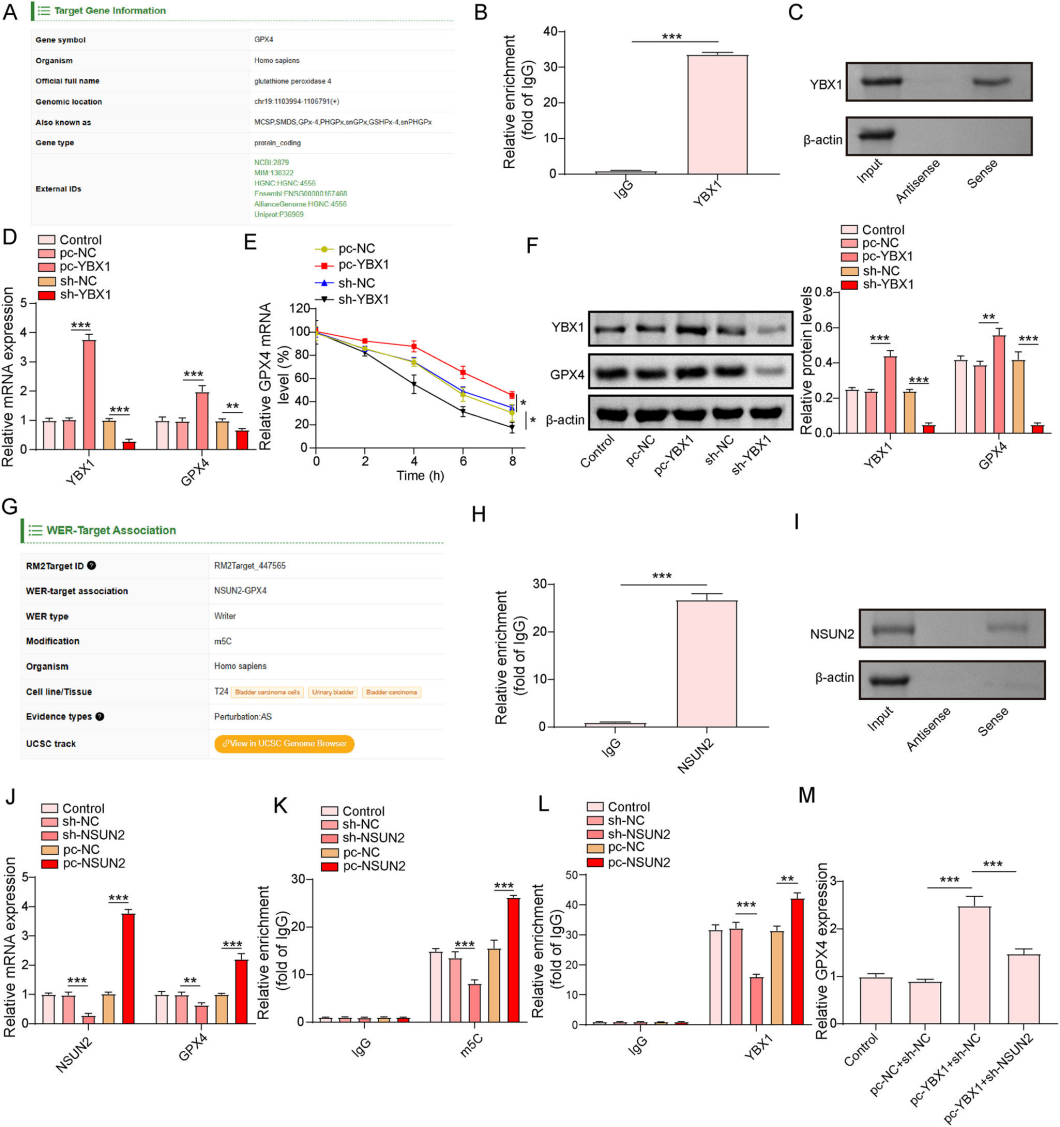
YBX1 enhanced the mRNA stability of GPX4. (A) RM2Target prediction of the binding relationship between YBX1 and GPX4 mRNA. (B, C) RIP nd RNA pull‐down confirming the YBX4–GPX4 protein‐R interaction in SH‐SY5Y cells. (D) The mRNA levels of YBX1 and GPX4 were quantified by RT‐qPCR after transfection with pcDNA3.1, pcDNA3.1‐YBX1, shNC, or shYBX1. (E) The effect of YBX1 on the stability of GPX4 mRNA was determined by Actinomycin D following YBX1 overexpression or knockdown. (F) Western blot analysis of YBX1 and GPX4 protein levels at 48 h post‐transfection after YBX1 overexpression or knockdown. (G) RM2Target prediction of NSUN2 targeting GPX4 mRNA. (H, I) RIP and RNA pull‐down validating NSUN2‐GPX4 association. (J) The mRNA levels of NSUN2 and GPX4 were validated by RT‐qPCR after shNSUN2 or shNC transfection, and upon NSUN2 overexpression (pc‐NSUN2 vs. pc‐NC). (K) m5C‐specific RIP assay detecting m5C modification levels on GPX4 mRNA following NSUN2 knockdown or overexpression. (L) YBX1 enrichment on GPX4 mRNA upon NSUN2 overexpression or knockdown was determined by RIP. (M) GPX4 mRNA levels in SH‐SY5Y cells co‐transfected with pc‐YBX1 and/or shNSUN2 was determined by RT‐qPCR. Data are mean ± SD from three independent experiments. *P < 0.05, **P < 0.01, and ***P < 0.001.

Having established that ETV4 transcriptionally up‐regulates YBX1, we asked how YBX1 feeds forward to preserve GPX4. RM2Target predicted the binding relationship between YBX1 and GPX4 (Figure 5A). This interaction was confirmed experimentally by RIP assay, which showed significant enrichment of GPX4 mRNA in the anti‐YBX1 antibody compared with the IgG (Figure 5B). Moreover, an RNA pull‐down assay using a biotinylated GPX4 probe efficiently retrieved YBX1 protein, demonstrating direct RNA‐protein binding (Figure 5C). Functionally, pc‐YBX1 transfection remarkably elevated mRNA levels of YBX1 and GPX4, while shYBX1 transfection down regulated YBX1 and GPX4 expression (Figure 5D). Actinomycin D treatment also revealed that YBX1 overexpression markedly prolonged and YBX1 knockdown shortened, the half‐life of GPX4 mRNA (Figure 5E), indicating that YBX1 stabilizes GPX4 mRNA. Furthermore, western blot analysis confirmed that YBX1 and GPX4 protein levels were increased by YBX1 overexpression and decreased by YBX1 knockdown (Figure 5F). Given that YBX1 often recognizes 5‐methyladenosine (m5C) ‐modified RNAs, we investigated the involvement of this RNA modification. RM2Target identified NSUN2, a key methyltransferase, as a writer of GPX4 mRNA (Figure 5G). RIP and RNA pull‐down verified that NSUN2 physically associated with GPX4 mRNA (Figures 5H and 5I). Furthermore, shNSUN2 transcription obviously reduced NSUN2 and GPX4 mRNA levels. In parallel, NSUN2 overexpression significantly increased NSUN2 and GPX4 mRNA levels (Figure 5J). Moreover, NSUN2 knockdown repressed the m5C enrichment on GPX4 mRNA, whereas NSUN2 overexpression markedly enhanced the m5C modification level of GPX4 mRNA (Figure 5K). To further establish whether YBX1 recruitment to GPX4 mRNA depends on NSUN2‐mediated m5C modification, we examined YBX1 enrichment on GPX4 mRNA upon NSUN2 overexpression or knockdown. The results revealed that, compared with the pc‐NC group, NSUN2 overexpression significantly enhanced the binding of YBX1 to GPX4 mRNA; conversely, compared with the shNC group, NSUN2 silencing markedly reduced YBX1 binding to GPX4 mRNA (Figure 5L). Importantly, the up regulation of GPX4 mRNA induced by YBX1 overexpression was significantly attenuated when NSUN2 was silenced (Figure 5M). The above data suggested that YBX1 stabilized GPX4 mRNA in an m5C dependent manner.

### 3.6 Knockdown of YBX1 Reversed the Biological Effects of ETV4 in OGD/R‐Induced Oxidative Stress and Ferroptosis

**FIGURE 6:**
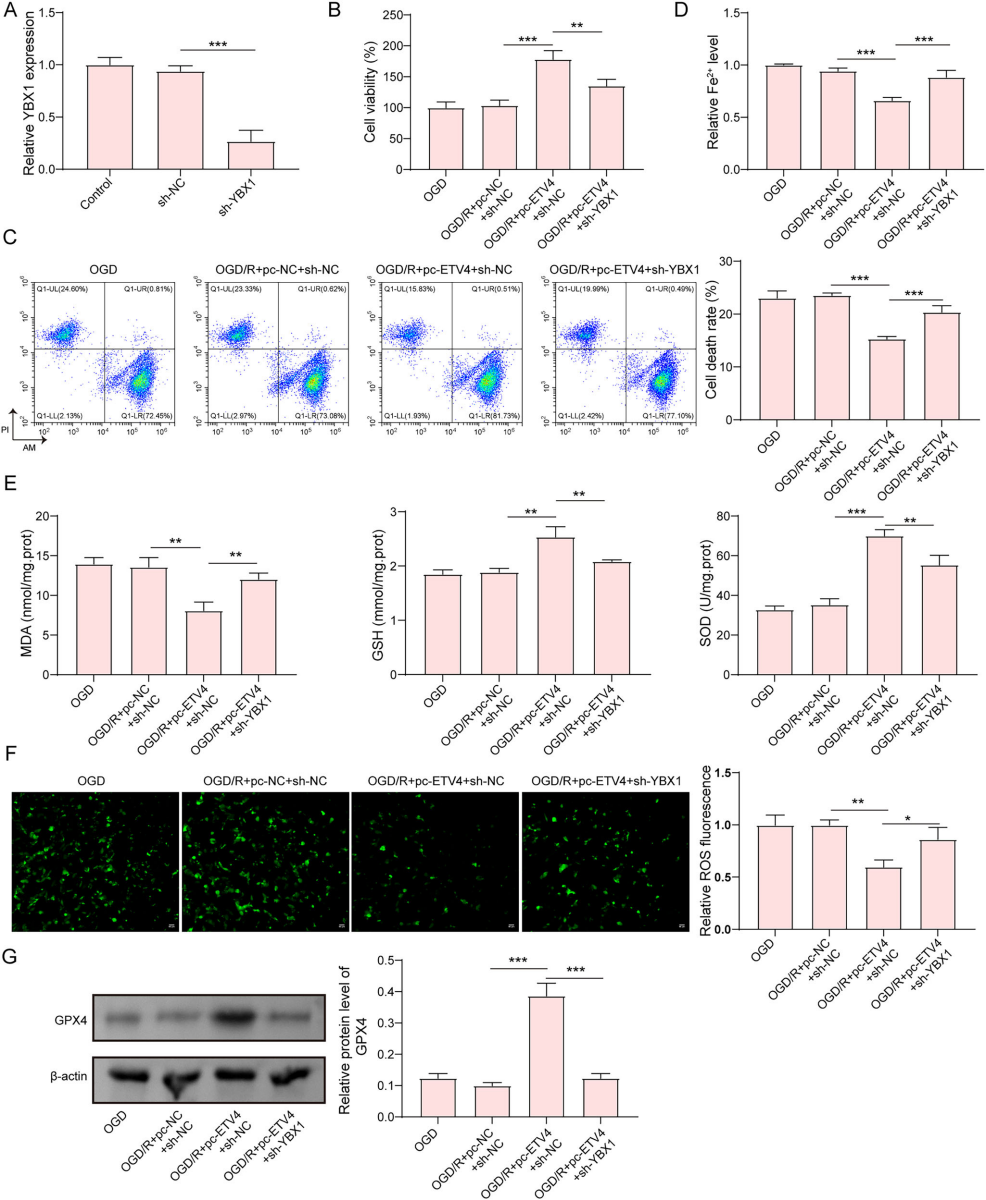
Knockdown of YBX1 reversed the biological effects of ETV4 in OGD/R‐induced oxidative stress and ferroptosis. (A) RT‐qPCR confirming the knockdown efficiency of YBX1 after transfection with shYBX1 vs shNC. SH‐SY5Y cells were transfected with pcDNA3.1‐ETV4 alone or co‐transfected with pcDNA3.1‐ETV4 and shYBX1, and then subjected to OGD/R; (B) CCK‐8 assay for cell viability; (C) Calcein‐AM/PI staining for assessing cell death rate; (D) ELISA assay for quantification of intracellular Fe^2^^+^ accumulation; (E) ELISA measurement for evaluating MDA, GSH and SOD levels; (F) DCFH‐DA staining for assessment of intracellular ROS levels; and (G) western blot was used to test GPX4 expression. Data are mean ± SD from three independent experiments. *P < 0.05, **P < 0.01, and ***P < 0.001.

To determine whether YBX1 was required for ETV4‐mediated protection against ferroptosis, YBX1 was knocked down in SH‐SY5Y cells using shRNA (Figure 6A). Then, functional experiments demonstrated that ETV4 overexpression significantly improved cell viability and reduced the cell death rate after OGD/R injury. However, these protective effects were largely abolished when YBX1 was knocked down (Figures 6B and 6C). Additionally, ETV4 overexpression attenuated OGD/R‐induced iron accumulation, and the elevation of MDA levels, as well as the decrease of SOD and GSH, whereas, these effects were abolished by YBX1 knockdown (Figures 6D and 6E). Similarly, the suppressive effect of ETV4 on ROS generation was also eliminated upon YBX1 silencing (Figure 6F). Finally, GPX4 protein was up regulated by pc‐ETV4 in OGD/R‐treated cells, and YBX1 silencing inhibited this facilitating effect (Figure 6G). Taken together, these data indicate that knockdown of YBX1 abrogated the anti‐oxidative stress and anti‐ferroptotic effect of ETV4 in SH‐SY5Y cells.

### 3.7 GPX4 Overexpression Reversed the Pro‐Ferroptotic Effects Induced by ETV4 Knockdown in OGD/R‐Treated SH‐SY5Y Cells

Here, we next investigated whether GPX4 mediates the protective effects of ETV4 against neuronal ferroptosis. SH‐SY5Y cells were transfected with a GPX4‐overexpressing plasmid (pc‐GPX4), and the up regulation of GPX4 mRNA and protein was confirmed by RT‐qPCR and western blot (Supplementary Figure 2A and 2B). Subsequently, cells were co‐transfected with shETV4 and either pc‐NC or pc‐GPX4, followed by exposure to OGD/R. As shown in Supplementary Figure 2C and 2D, GPX4 overexpression significantly ameliorated the reduction in cell viability and the increase in the cell death rate caused by ETV4 knockdown under OGD/R conditions. Moreover, the exacerbated Fe^2^^+^ accumulation, MDA elevation, and GSH and SOD depletion induced by ETV4 silencing were markedly reversed by GPX4 restoration (Supplementary Figure 2E and 2F). GPX4 overexpression also attenuated excessive ROS production resulting from ETV4 deficiency in OGD/R‐treated cells (Supplementary Figure 2G). Western blot analysis confirmed that GPX4 protein levels, which were reduced upon ETV4 knockdown, were effectively restored by pc‐GPX4 transfection (Supplementary Figure 2H). Collectively, these findings suggest that GPX4 overexpression counteracts the pro‐ferroptotic effects of ETV4 silencing, indicating that ETV4 exerts its neuroprotective function at least in part through regulating GPX4‐mediated ferroptosis.

## 4 Discussion

CIRI represents a critical pathological process with profound clinical implications, often leading to severe neurological deficits, and neuronal death has been recognized as a central event driving its progression (Li et al. 2022). In particular, ferroptosis, an iron‐dependent form of regulated cell death characterized by lipid peroxidation, has recently emerged as a key mechanism contributing to neuronal loss in CIRI, highlighting its significance as a potential therapeutic target (Li et al. 2021). In this study, we systematically investigated the molecular mechanisms underlying ferroptosis in neuronal models of CIRI. Our findings demonstrated that ETV4 enhanced the transcriptional up regulation of YBX1, which in turn stabilized GPX4 mRNA through m5C‐mediated modification. This process effectively inhibited neuronal ferroptosis and attenuated CIRI. The elucidation of ETV4 not only deepens our understanding of the regulatory network governing ferroptosis in CIRI but also provides a new theoretical basis for developing targeted interventions aimed at mitigating ferroptosis and improving functional recovery after CIRI.

ETV4, a member of the PEA3 subfamily of ETS transcription factors, which acts as a critical regulator of neuronal development and brain injury‐related diseases. For example, reactivating ETV4 restores VGLUT3 expression, rescues synaptic transmission, and rescues memory performance (He et al. 2022). Loss of ETV4 disrupts hippocampus‐dependent cognition (Fontanet et al. 2018). ETV4 is an essential downstream effector that links BDNF/TrkB/ERK1/2 signaling to VGLUT3 transcription and axonal growth, highlighting its potential role in sensory neuron regeneration and repair (Liu et al. 2016). We previously reported that BMSC‐derived exosomal lncRNA KLF3‐AS1 mitigates CIRI through interaction with ETV4, which promotes the transcriptional up regulation of Sirt1 and subsequently augments autophagic activity (Xu et al. 2023). Emerging evidence has begun to link ETV4 to the regulation of ferroptosis. For instance, the activation of ETV4/SLC7A11/GPX4 cascade has been shown to suppress ferroptosis in chondrocytes, thereby ameliorating osteoarthritis (He et al. 2024). However, the functional role and underlying mechanism of ETV4 in neuronal ferroptosis remain entirely unknown. In the present study, we provide the first evidence that ETV4 serves as an endogenous suppressor of ferroptosis in the setting of CIRI. We found that ETV4 was rapidly down regulated in both an OGD/R neuronal model and the MCAO/R rat brain, and that restoring its expression effectively blocked the iron‐overload and oxidative injury, key characteristics of ferroptosis. Collectively, these data position ETV4 as a druggable node for ferroptosis‐targeted neuroprotection in CIRI. Beyond ferroptosis, ETV4 has been implicated in the coordinated control of apoptosis, inflammation, and autophagy across various pathological contexts. In colorectal cancer, ETV4 suppresses autophagy via the P300/ETV4‐WDR4 axis (Deng et al. 2025), whereas its knockdown promotes autophagy‐dependent apoptosis in glioblastoma (Wang et al. 2022). METTL3‐driven m6A enhancement of ETV4 stability drives NF‐κB‐mediated neuroinflammation and caspase‐3‐dependent apoptosis in microglia (He et al. 2025). Additionally, ETV4 directly activates TNF‐α transcription to drive hepatic inflammation (Qi et al. 2023). In the context of CIRI, ETV4 can enhance autophagy and inhibit microglial apoptosis by up regulating SIRT1 (Xu et al. 2023), suggesting that ETV4 may play a multifaceted protective role in CIRI that extends beyond a single cell death pathway.

YBX1 is an evolutionarily conserved, multifunctional nucleic‐acid‐binding protein that operates as a master post‐transcriptional regulator of gene expression, stress granule assembly and DNA‐damage repair (Zheng et al. 2025). Several studies have interrogated its role in CIRI. For instance, YBX1 has been shown to promote PINK1/Parkin‐dependent mitophagy, inhibit neuronal pyroptosis and apoptosis, thereby reducing infarct volume and improving neurological outcomes (Peng et al. 2025; Tuerxun et al. 2021; Li et al. 2024). Additionally, YBX1 has been reported to inhibit ferroptosis in osteoblasts under iron‐overload conditions (Tong et al. 2025), and to attenuate ferroptosis‐driven myocardial injury during diabetic ischaemia‐reperfusion (Li et al. 2025). These findings suggest that YBX1 might also counteract neuronal ferroptosis in CIRI. Our study unveiled a novel transcriptional mechanism through which ETV4 conferred protection against ferroptosis by directly activating YBX1 expression. This finding not only identified ETV4 as an upstream transcriptional regulator of YBX1 but also establishes a mechanistic bridge linking ETV4 to the antioxidant machinery responsible for ferroptosis suppression. In addition, YBX1 knockdown abrogated the protective effects of ETV4 overexpression, reinstating oxidative stress, and iron accumulation. These results firmly establish a linear causal relationship within the ETV4/YBX1/GPX4 axis in CIRI.

As a critically significant form of methylation, m5C modification exerts profound influences on RNA through the regulation of transcriptional activity, structural configuration, and stability (Bohnsack et al. 2019). The functional significance of m5C RNA modifications is increasingly recognized in neurological disorders and brain injury contexts, including CIRI (Wu et al. 2024). For instance, TET1‐dependent m5C methylation of RelB exacerbates neuroinflammation following cerebral ischemia/reperfusion (I/R) by modulating microglial polarization (Lin et al. 2024). Additionally, dynamic alterations in neuronal mRNA m5C profiles have been documented under OGD/R conditions (Jian et al. 2021), underscoring the potential involvement of m5C in post‐ischemic RNA regulation. YBX1 acts as a canonical m5C “reader” protein, recognizing m5C‐modified transcripts via its conserved cold‐shock domains to facilitate RNA stability and translation (Meng et al. 2024, Li et al. 2024, Li et al. 2025). In this study, we demonstrated that YBX1 specifically binds to m5C‐marked GPX4 mRNA, thereby protecting it from degradation and sustaining GPX4 protein synthesis—a crucial brake on ferroptosis. This mechanism highlights YBX1's role as a post‐transcriptional effector in the ETV4/YBX1/GPX4 axis. Notably, the m5C modification on GPX4 mRNA was catalyzed by the methyltransferase NSUN2, a key writer of m5C with established roles in various physiological and pathological processes, including tumorigenesis (Li and Huang 2024). A recent study reported that NSUN2 activation promoted m5C‐dependent astrocytic neuroinflammation, thus exacerbating CIRI (Wang et al. 2026). Our study extends this understanding by revealing that NSUN2‐mediated m5C methylation of GPX4 mRNA is essential for its recognition and stabilization by YBX1, thereby elucidating a novel NSUN2/m5C/YBX1/GPX4 regulatory axis that constrains neuronal ferroptosis and may open new avenues for therapeutic intervention in CIRI.

In conclusion, this study unveils that ETV4 transcriptionally activates YBX1, which stabilizes GPX4 mRNA by binding NSUN2‐installed m5C modification, thereby inhibiting ferroptosis in CIRI. Therefore, targeting the ETV4/YBX1 axis may represent a promising therapeutic strategy for CIRI, highlighting its clinical importance in mitigating ischemic brain injury. This study had several limitations. For example, ETV4 may regulate additional targets in regulating ferroptosis after CIRI, which needs further exploration. Additionally, although TUNEL staining was used to assess cell death, it remains unclear whether ETV4 confers protection specifically against ferroptosis or additionally modulates other cell death pathways such as apoptosis; thus, future studies examining cleaved Caspase‐3 or other apoptotic markers will be important to clarify the broader protective mechanisms of ETV4 beyond ferroptosis. Furthermore, further studies are required to validate the mechanism and to explore the clinical application. Furthermore, extend the paradigm to other ferroptosis‐driven pathologies, such as intracerebral haemorrhage, traumatic brain injury, Parkinson's disease, and tumorigenesis, to determine whether the ETV4‐YBX1‐GPX4 axis represents a universal anti‐ferroptosis function.

## Author Contributions

**Qian Xu**: conceptualization, methodology, data curation, investigation, writing – original draft, writing – review and editing, software, validation. **Faming Deng**: investigation, data curation, writing – review and editing, visualization. **Dan Yu**: writing – review and editing, methodology, formal analysis, supervision, resources.

## Funding

The authors have nothing to report.

## Ethics Statement

All experimental procedures were approved by the Animal Ethics Committee of Haikou Affiliated Hospital of Central South University Xiangya School of Medicine. The procedures were performed in accordance with the ARRIVE guidelines.

## Consent

The authors have nothing to report.

## Conflicts of Interest

The authors declare no conflicts of interest.

## Supporting information




**Supplementary Figures**: brb371548‐supp‐0001‐FigureS1‐S2.docx

## Data Availability

The data that support the findings of this study are available from the corresponding author upon reasonable request.
