# Supplementary material for: ETV4 Improves Cerebral Ischemia‐Reperfusion Injury by Restraining YBX1‐GPX4‐Ferroptosis Cascades
Source: Brain Behav. 2026 Jun 16;16(6):e71548. doi: 10.1002/brb3.71548 (PMC13272628; doi:10.1002/brb3.71548)
Supplement: Supplementary file 1 — Supplementary Figures: brb371548‐supp‐0001‐FigureS1‐S2.docx [file BRB3-16-e71548-s001.docx]

**Supplementary figure legends**

**Supplementary Figure 1.** ETV4 knockdown aggravated OGD/R-induced oxidative stress and ferroptosis in SH-SY5Y cells. (A, B) SH-SY5Y cells were transfected with shNC, sh-ETV4#1, or sh-ETV4#2. ETV4 mRNA and protein levels were measured using RT-qPCR and Western blot to verify knockdown efficiency. SH-SY5Y cells were transfected with shNC or shETV4 for 48 h, followed by exposure to OGD/R. (C) CCK-8 assay for cell viability. (D) Calcein-AM/PI staining for determining cell death rate. (E) ELISA quantification of intracellular Fe²⁺ accumulation. (F) ELISA measurement of MDA, GSH and SOD levels. (G) DCFH-DA assessment of intracellular ROS level. (H) Western blot was used to test GPX4 expression. Data are presented as mean ± SD from three independent experiments. *P<0.05, **P<0.01, ***P<0.001.

**Supplementary Figure 2.** GPX4 overexpression reversed the pro-ferroptotic effects induced by ETV4 knockdown in OGD/R-treated SH-SY5Y cells. (A, B) SH-SY5Y cells were transfected with pc-NC or pc-GPX4. GPX4 mRNA and protein levels were measured using RT-qPCR and Western blot to verify overexpression efficiency. SH-SY5Y cells were transfected with the indicated plasmids for 48 h, followed by exposure to OGD/R. (C) CCK-8 assay for cell viability. (D) Calcein-AM/PI staining for determining cell death rate. (E) ELISA quantification of intracellular Fe²⁺ accumulation. (F) ELISA measurement of MDA, GSH and SOD levels. (G) DCFH-DA assessment of intracellular ROS level. (H) Western blot was used to test GPX4 expression. Data are presented as mean ± SD from three independent experiments. *P<0.05, **P<0.01, ***P<0.001.
